# Thermal Sensors for Contactless Temperature Measurements, Occupancy Detection, and Automatic Operation of Appliances during the COVID-19 Pandemic: A Review

**DOI:** 10.3390/mi12020148

**Published:** 2021-02-03

**Authors:** Elisabetta Moisello, Piero Malcovati, Edoardo Bonizzoni

**Affiliations:** Department of Electrical, Computer and Biomedical Engineering, University of Pavia, via Ferrata 5, 2100 Pavia, Italy; piero.malcovati@unipv.it (P.M.); edoardo.bonizzoni@unipv.it (E.B.)

**Keywords:** bolometer, COVID-19, motion, occupancy, pyroelectric sensor, temperature, thermal detector, thermal sensor, thermopile, TMOS

## Abstract

The worldwide spread of COVID-19 has forced us to adapt to a new way of life made of social distancing, avoidance of physical contact and temperature checks before entering public places, in order to successfully limit the virus circulation. The role of technology has been fundamental in order to support the required changes to our lives: thermal sensors, in particular, are especially suited to address the needs arisen during the pandemic. They are, in fact, very versatile devices which allow performing contactless human body temperature measurements, presence detection and people counting, and automation of appliances and systems, thus avoiding the need to touch them. This paper reviews the theory behind thermal detectors, considering the different types of sensors proposed during the last ten years, while focusing on their possible employment for COVID-19 related applications.

## 1. Introduction

Thermal sensors can be employed in a wide range of applications such as gas analysis, pedestrian detection for autonomous driving, car climate control, car seat occupancy, space and military applications, security systems, medical devices, temperature monitoring in manufacturing processes, appliances and consumer products (microwave ovens, clothes driers, laser printers) [1,2,3,4,5,6,7,8,9,10,11]. In particular, as they respond to thermal radiation, they are especially suited for implementing many contactless applications, which have become increasingly widespread in recent times as a way to face the COVID-19 pandemic. In order to limit the virus circulation, indeed, contactless temperature measurements before entering public offices, restaurants and shops have become normal, as well as the automation of appliances (e.g., sanitizing gel dispensers, lights, heating, ventilation and air conditioning systems) to avoid touching them. Furthermore human presence detection, enabling people counting, could be employed to monitor occupancy in public spaces to ensure that social distancing policies are respected without affecting people’s privacy. Low-power, low-cost sensor solutions are of particular interest as, after the emergency situation due to the coronavirus pandemic will have passed, they could also be employed in the growing market of smart homes, Internet of Things (IoT) and wearable devices.

Thermal sensors are based on a temperature change of the detector element through the absorption of radiation from a target object: the temperature change determines in turn a change in a temperature-dependent property of the sensor, which is evaluated electrically and gives a measure of the absorbed radiation from the target, from which its temperature can be derived. Thermal sensors, in fact, exploit the Stefan–Boltzmann law, which states that every object emits thermal radiation depending on its temperature [12,13]. In the case of the human body, the emitted radiation falls within the infrared (IR) range, peaking at around 12 µm wavelength.

There exist different types of thermal sensors: bolometers, pyrometers, thermopiles and micromachined CMOS transistors, from now on referred to as “TMOS”. In this paper, the different detector technologies will be presented, analyzing their principle of operation and comparing them in order to highlight advantages and drawbacks. Sensor solutions proposed during the last ten years have been taken into consideration, focusing on those which are or could be employed in applications in the fight against the COVID-19 pandemic.

The paper is organized as follows: Section 2 illustrates the theory behind the different types of thermal sensors, Section 3 reviews the state-of-the-art for the illustrated detector types, while Section 4 concludes the paper, providing a comparison among the different considered thermal sensors, taking into account their employment in applications helpful for facing the COVID-19 pandemic.

## 2. Thermal Detectors Theory

Thermal sensors respond to the thermal radiation emitted by any object located within the solid angle determined by their field-of-view (FOV) by producing an electric signal (typically voltage), that is proportional to the incident power. The FOV is defined as the difference of the incidence angles that allow the sensor to receive 50% relative output signal. The fundamental figure-of-merit for characterizing a thermal detector is, therefore, its responsivity [14], defined as

(1) $$R=\frac{V_{out}}{P_{in}}$$

where $V_{out}$ is the sensor output voltage signal and $P_{in}$ the incident radiant power falling on the detector, which corresponds to the net power exchange between the detector and the radiation source.

Knowing the detector and source temperatures, $T_{d}$ and $T_{s}$, the thermal sensor responsivity R and the net power exchange from the source $P_{in}$, therefore, the output signal $V_{out}$ can be estimated as follows:

(2) $$V_{out}\left(T_{d},T_{s}\right)=R\;P_{in}\left(T_{d},T_{s}\right)$$

$P_{in}$, and therefore $V_{out}$, depend on several factors, apart from the detector and the source object temperatures: the detector and source emissivity, the presence of additional objects in the path (e.g., optics), the shape and area of detector and source, as well as the orientation and the distance between them, the detector FOV and the medium between detector and source object.

Taking into consideration all these factors and supposing that the medium is air and that the intrinsic FOV of the detector is not reduced (i.e., the source object is seen in its entirety by the detector), $P_{in}$ can be expressed as

(3) $$P_{in}\left(T_{d},T_{s}\right)=\frac{\sigma \epsilon_{s}\epsilon_{d}A_{s}F_{sd}}{\pi }\left(T_{s}^{4}-T_{d}^{4}\right)$$

where σ is the Stefan-Boltzmann constant, equal to 5.67 $\times 10^{-12}$$\frac{W}{{\mathrm{cm}}^{2}K^{4}}$, $\epsilon_{s}$ the source object emissivity, $\epsilon_{d}$ the detector emissivity, $A_{s}$ the source area and $F_{sd}$ a transfer factor which takes into account the detector-source system geometry [15].

**Figure 1:**
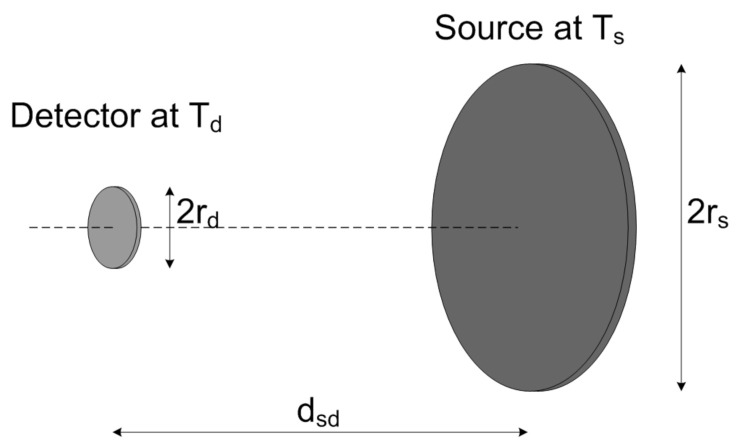
Schematic representation of the considered detector-source object system geometry [15].

Assuming the geometrical configuration illustrated in Figure 1, where detector and source are coaxial disks, the transfer factor $F_{sd}$ is given by

(4) $$F_{sd}=\frac{2\pi r_{d}^{2}}{r_{s}^{2}+r_{d}^{2}+d_{sd}^{2}+\sqrt{{\left(r_{s}^{2}+r_{d}^{2}+d_{sd}^{2}\right)}^{2}-4r_{s}^{2}r_{d}^{2}}}$$

where $r_{d}$ is the detector radius, $r_{s}$ the source object radius and $d_{sd}$ the distance between detector and source [15]. The relationship given by Equation (4) still holds if the detector and the target object are rectangular in shape: in this case a circular detector and source object of equivalent area must be considered.

**Figure 2:**
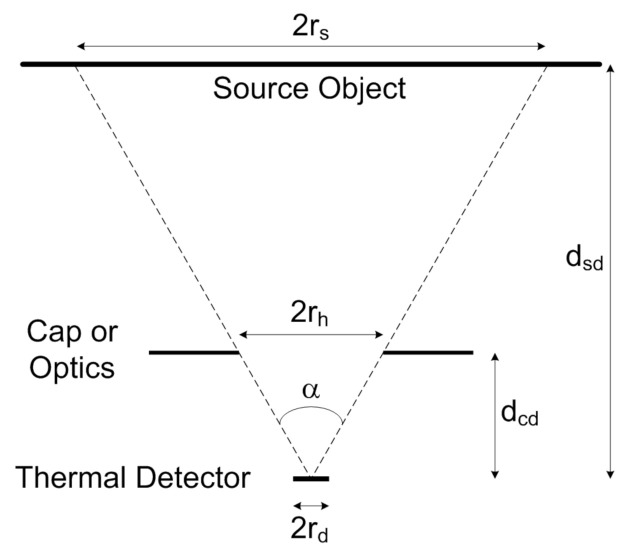
Schematic representation of the sensor FOV angle [15].

If the sensor FOV is limited (e.g., by a perforated cap or by optics), as illustrated in Figure 2, the FOV angle is calculated, relying on trigonometry, as:

(5) $$\alpha =2\;arctan\left(\frac{r_{h}}{d_{cd}}\right)$$

where $r_{h}$ is the aperture radius and $d_{cd}$ the distance of the cap from the detector.

Provided that the target object fills the entire detector FOV, the model described by Equations (2)–(4) still holds, with $A_{s}$ = $\pi r_{s}^{2}$ and $r_{s}$ given by:

(6) $$r_{s}=d_{sd}\;tan\left(\frac{\alpha }{2}\right)$$

If the FOV is completely filled by the target object, the incident radiant power falling on the detector exhibits almost no dependence on the distance between the sensor and the radiator. In fact, considering the modelled thermal detector output signal and applying Equation (6), provided that, as it is verified in the targeted practical applications, $r_{d}\ll d_{sd}$, the following expression holds true:

(7) $$F_{sd}\;A_{s}\approx \frac{2\;d_{sd}^{2}\;{tan}^{2}\left(\frac{\alpha }{2}\right)\;r_{d}^{2}\;\pi^{2}}{2\;d_{sd}^{2}\;\left({tan}^{2}\left(\frac{\alpha }{2}\right)+1\right)}=\frac{{tan}^{2}\left(\frac{\alpha }{2}\right)\;r_{d}^{2}\;\pi^{2}}{{tan}^{2}\left(\frac{\alpha }{2}\right)+1}$$

All thermal detectors feature an absorbing membrane in order to collect radiation from the target object and the surroundings. The membrane is designed in order to be sensitive to the desired radiation wavelength interval, i.e., IR around 12 µm wavelength for human body temperature measurements, occupancy detection, people counting and appliances automation applications. To correctly absorb the radiation, the membrane has to be properly thermally isolated: for this purpose, micromachining techniques are usually employed, in order to release the sensor and obtain a suspended structure with high thermal isolation and low thermal mass.

The thermal characteristics of the sensor, i.e., its effective thermal conductance $G_{th}$ and heat capacitance $C_{th}$, determine its response time τ as

(8) $$\tau =\frac{G_{th}}{C_{th}}$$

The sensor response time characterizes the sensor transient response when the input IR power changes abruptly and consists of the time required for the transient output signal to reach $2^{-1/2}$ of its steady-state value. For thermal detectors, τ ranges between 0.001 s and 0.1 s: thermal sensors, therefore, are suitable for detecting human motion and presence, as well as for realizing almost instantaneous absolute temperature measurements.

Thermal detectors suffer from noise, both electronic and thermal. Electronic noise is determined by the detector resistance (bolometers and thermopiles) or by the transistor noise (TMOS), while thermal noise is due to environmental disturbances.

Typically, thermal detectors are packaged under vacuum in order to reduce the thermal losses due to conduction, thus increasing their performance.

These theoretical models and figures-of-merit are applicable to every type of thermal detector. The principle of operation of each thermal detector type will be discussed in the following paragraphs.

### 2.1. Bolometers

Bolometers and their miniaturized version (microbolometers) consist of an absorbing layer, implementing the membrane that collects radiation, an active layer, that acts as an embedded resistor whose resistance value varies with temperature, and support arms which provide electrical connection to the circuitry while maintaining the sensor suspended and thermally isolated. The incident IR radiation absorbed by the bolometer membrane warms up the embedded resistor and as a result, its resistance is changed. The resistance variation is expressed by the temperature coefficient of resistance

(9) $$TCR=\frac{1}{R}\frac{dR}{dT}$$

where *R* is the resistance and *T* the temperature [16]. The temperature coefficient of resistance (TCR) can be either positive or negative.

The variation in the resistance, ΔR, determines in turn a voltage variation which, according to Ohm’s law, is

(10) ΔV=IΔR=IRTCRΔT

where *I* is the bolometer biasing current. Biasing is necessary in order to allow the measurement of the resistance variation.

### 2.2. Pyrometers

**Figure 3:**
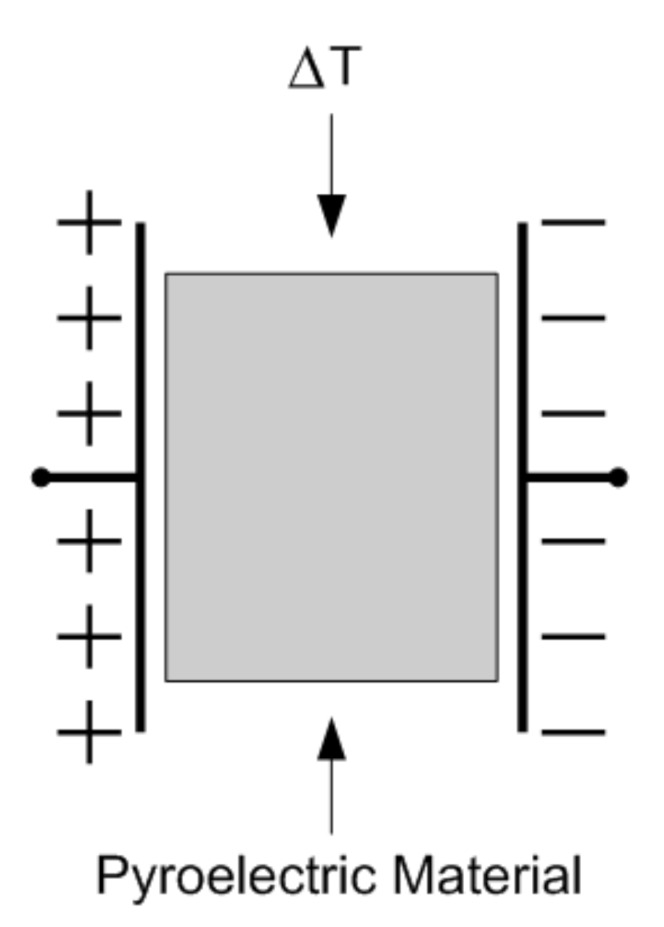
Conceptual representation of a pyroelectric material.

Pyroelectric detectors are made of crystals which display a variation in polarization depending on their temperature: when the crystal temperature changes, charges originate on its faces, as illustrated in the conceptual representation of Figure 3. This mechanism, known as pyroelectric effect, is analogous to the one that occurs in piezoelectric materials when a mechanical stress is applied. The pyroelectric effect is expressed through the so-called pyroelectric coefficient

(11) $$p=\frac{dP}{dT}$$

where *P* is the polarization and *T* the temperature [16].

In the presence of changes in the temperature, the variation in the polarization and the resulting variation in the charge *Q* can be, therefore, expressed in terms of the pyroelectric coefficient as follows

(12) P=pΔT

(13) Q=pAT

where *A* is the detector area.

The variation in the charge causes a current

(14) $$I=\frac{dQ}{dt}=A\;p\;\frac{dT}{dt}$$

Pyroelectric detectors thus display a current transient in response to temperature variations and need a constant current biasing: they are AC devices and cannot provide measurements at DC.

### 2.3. Thermopiles

**Figure 4:**
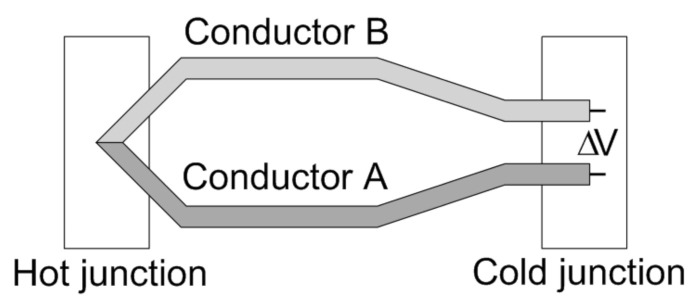
Conceptual representation of a thermocouple [22].

Thermopile sensors consist of *N* thermocouple elements placed in series: this allows increasing the sensor output voltage signal to *N* times the one of a single thermocouple. Thermocouple elements consist of two different conductor materials joined at one end. The joined end in denominated hot junction, while the other end is called cold junction, as indicated in the schematic view of Figure 4. When the two junctions are at different temperatures, thanks to the Seebeck effect [17,18], a voltage difference ΔV originates between the two conductor materials:

(15) ΔV=αΔT

where ΔT is the temperature difference between the hot and the cold junction and α is the Seebeck coefficient, which depends on the employed conductor materials.

**Figure 5:**
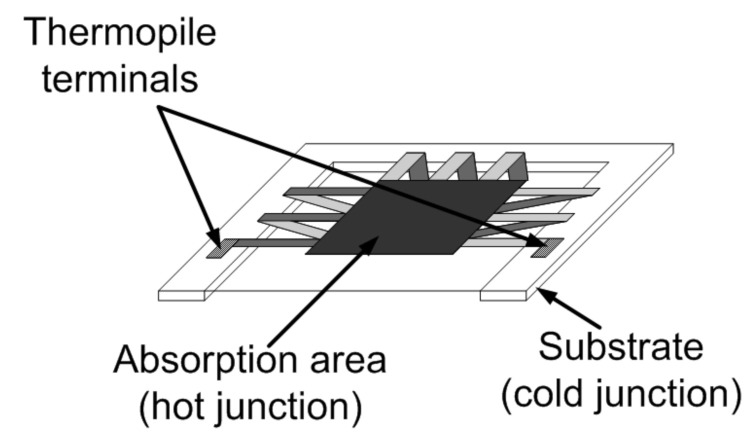
Conceptual representation of a micromachined thermopile sensor [22].

In a micromachined thermopile, as illustrated in Figure 5, the hot junctions are embedded in the absorbing membrane, while the cold junctions are located on the substrate. The absorbed incident thermal radiation from the target object determines a heating of the membrane and then a temperature difference between the two junctions, which gives rise to the voltage signal.

Thermopiles, therefore, measure a temperature difference between the junctions. Hence, in order to perform absolute temperature measurements, the temperature of the cold junction should be known, for example employing an integrated $V_{BE}$-based sensor [19,20,21].

Thermopiles are self-powered and thus do not require any biasing.

### 2.4. TMOS

The TMOS has been developed in the last decade thanks to the work of the Technion-Israel Institute of Technology in Haifa, Israel [23,24,25]. This novel type of thermal sensor is based on a micromachined thermally isolated transistor, fabricated in a standard CMOS-SOI process, embedded in a membrane able to absorb IR radiation from a target object, inducing a variation of the transistor temperature and, therefore, generating a signal by changing the transistor I–V characteristics. SOI (Silicon-On-Insulator) technology is preferred over regular CMOS processes, as it allows achieving good thermal isolation more easily.

With respect to conventional thermal detectors (i.e., bolometers, pyrometers and thermopiles), as the TMOS is an active sensig element, it features advantages in terms of internal gain, resulting in high responsivity and sensitivity, which make the TMOS particularly appealing.

The TMOS performance depends on the transistor operating region, as well as on its configuration (two terminals diode-like, three terminals) [26]. Subthreshold region is preferred as operating region for the TMOS as it yields the largest sensitivity value [23,27]. In subthreshold region, in fact, the transistor current operation is based on diffusion and, therefore, more sensitive to temperature. Furthermore, lower noise is contributed by the transistor and self-heating effects are drastically reduced [28].

Being an active device, the TMOS requires biasing; however, when employed in subthreshold region, it can feature very low power consumption.

The TMOS signal can be modeled as a temperature dependent current source, $i_{sig}$, in parallel with the $g_{m}V_{gs}$ generator in the small signal equivalent circuit [24].

The temperature dependent current source is defined as

(16) $$i_{sig}=\Delta T_{TMOS}\frac{dI_{DS}}{dT}$$

where $\frac{dI_{DS}}{dT}$ is the transistor drain-to-source current variation with respect to the TMOS temperature for the considered operating point and Δ$T_{TMOS}$ is the temperature variation induced on the TMOS sensor by the radiation absorbed from the target object, which is defined as

(17) $$\Delta T_{TMOS}=\eta \frac{P_{in}}{G_{th}}$$

where η is the absorbing efficiency, $P_{in}$ the incident radiation power and $G_{th}$ the sensor thermal conductance [24].

The TMOS can be operated either in current or voltage mode [29]. Depending on the operation mode, the TMOS sensitivity can be expressed either with the temperature coefficient of current (TCC), for current mode, or with the temperature coefficient of voltage (TCV), for voltage mode. TCC and TCV, similarly to the TCR of bolometers, are defined as

(18) $$TCC=\frac{1}{I_{DS}}\frac{dI_{DS}}{dT}$$

(19) $$TCV=\frac{1}{V}\frac{dV}{dT}$$

where $I_{DS}$ is the transistor drain-to-source current and *V* its voltage bias.

## 3. State-of-the-Art Review

### 3.1. Bolometers

Bolometers are widely used in military and marine systems, automotive, thermography and surveillance. However, they are still too expensive for allowing their widespread use in consumer products and applications where cost is a constraint, as in the mass production of sensors to be used for COVID-related applications. Although they feature good temperature sensitivity properties, in fact, the materials employed to realize the embedded resistor, such as vanadium oxide [30,31] or amorphous silicon [31,32], require complex post-CMOS processes, lithography and material deposition [33].

The recent trends in research, therefore, have been focused not only on improving bolometers performance, but also on designing bolometers which could be fabricated at a lower cost. For example, the cost of bolometers could be substantially reduced if they were produced in high volume CMOS/MEMS (Micro-Electrical Mechanical Systems) foundries [34].

Microbolometers employing vanadium oxide were presented in [35]: instead of relying on thin micromachined membranes to provide thermal insulation from the substrate, the approach consisted in fabricating the resistors acting as thermistors on thin thermally insulating polyimide substrates. This solution ensures simplicity and low cost and allows realizing more robust and reliable devices; however, it faces the main challenge of producing high quality and high sensitivity devices on thin films which are non-planar, imperfect and porous substrates.

**Figure 6:**
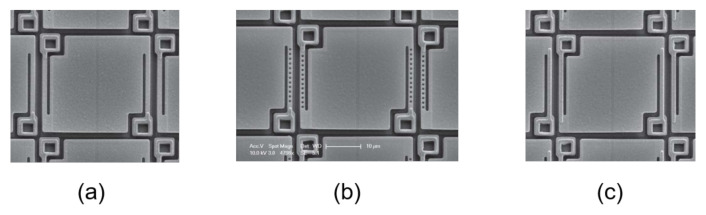
Microphotograph of the (**a**) conventional, (**b**) meshed leg and (**c**) extended leg bolometer sensors presented in [31].

With respect to vanadium oxide, amorphous silicon is a more optimized material for thermal IR detectors, as it features a smaller resistance value which results in a better responsivity property [32]. In order to improve amorphous silicon-based sensors, different solutions have been considered for reducing the pixel pitch size and, therefore, the cost, while improving resolution and productivity. Diminishing the pixel pitch, however, degrades the detector sensitivity as the pixel fill factor is reduced: the legs sustaining the suspended structure, in fact, are not scaled accordingly with the pixel pitch, as they cannot be too small in order to avoid bending of the pixel structure and deterioration of resistance uniformity. Different solutions, therefore, have been considered in [31] for optimizing the leg structure while reducing the pixel pitch size and improving the fill factor. While maintaining the same leg width and length, one solution features holes to form a meshed leg structure, while the other employs an additional metal layer to increase the leg thickness: in this way, the leg structures are designed to change the thermal time constant by varying the thermal conductance. The conventional, meshed leg and extended leg solutions are illustrated in Figure 6.

**Figure 7:**
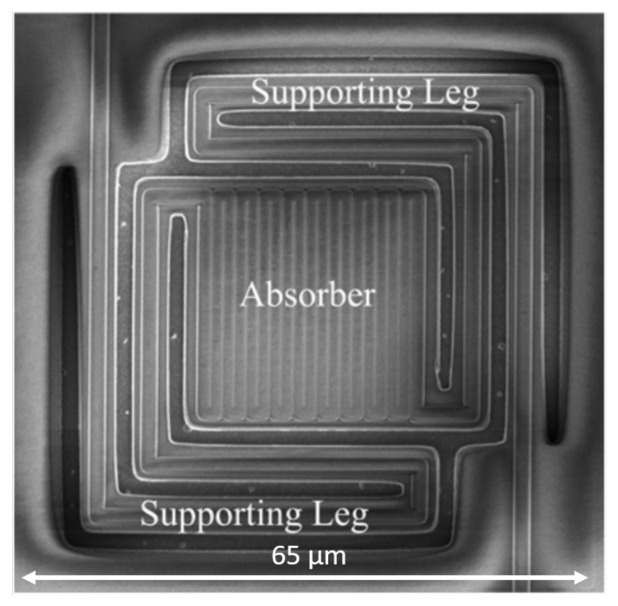
Microphotograph of the bolometer sensor presented in [33].

In order to reduce the fabrication cost of the detector, microbolometer implementations in standard CMOS processes have been investigated in the literature. In [33], a low-cost double-sacrificial layer microbolometer realized in a standard 0.5-µm CMOS process is presented: in this instance, the CMOS metal interconnect layer, made of aluminum (Al), is used as the IR sensitive material. The resulting TCR value, however, equal to 0.385%/K, is too low for many applications. This microbolometer is illustrated in the microphotograph of Figure 7.

In order to lower the design and fabrication complexity and, consequently, the cost of the detector, a solution is employing the same material for the absorbing, active and structural layers, realizing a single layer detector. In [36], a single layer microbolometer was implemented employing platinum film; however, the TCR equal to 0.14/%K is too low for high performance applications, such as for example contactless fever measurements. In [37], instead, zinc oxide (ZnO) was employed for realizing the single layer microbolometer: ZnO features a TCR value of −10.4/%K, significantly larger than common microbolometer materials [38,39], while also having high absorption characteristics and low internal stress, thus resulting particularly suitable as active, as well as absorbing and structural, material.

In order to improve microbolometers performance, different materials, in addition to the already mentioned ones, have recently been studied: titanium and titanium oxide, yttrium barium copper oxide, Si/SiGe [40,41,42,43] and organic materials such as cytochrome C protein [44,45]. Titanium and titanium oxide, yttrium barium copper oxide and Si/SiGe, however, still require complex post-CMOS process steps; cytochrome C protein, instead, can be deposited on sensing pixels using spin coating [45] or an inject printer [44], thus avoiding time-consuming vacuum deposition and expensive photolithography processes. In [44,45], a microbolometer consisting of suspended aluminum electrodes covered by a cytochrome C thin film is presented: it features a 28–29%/K TCR, significantly larger than typical bolometer materials. This advantage, together with the limited production cost, however, comes at the price of a reduced stability with respect to non-organic materials, which can be addressed by the appropriate choice of packaging [45], that can however reduce the savings allowed by the cheaper production steps.

Table 1 reports the main characteristics of various microbolometers types. The considered bolometers, in the referenced papers, featured a general characterization of their properties [32,33,37,44] or were tested as the unit pixel of an IR imager matrix [30,31]; however, they could all be employed for motion and presence detection, at least in a short distance range, thus enabling people counting and appliances automation applications. In order to be suitable for contactless fever measurements, the featured sensor noise equivalent temperature difference (NETD), and therefore its resolution, must be lower than 0.1–0.3 °C: this requirement is satisfied by the bolometers presented in [30,37,44] and the meshed leg type in [31].

Bolometers, therefore, thanks to their high TCR, are suitable for contactless human body temperature measurements, as well as for presence and motion detection applications. Their main disadvantage is given only by their fabrication cost, as typically employed bolometer materials require expensive process steps on top of the regular CMOS technology.

### 3.2. Pyrometers

**Figure 8:**
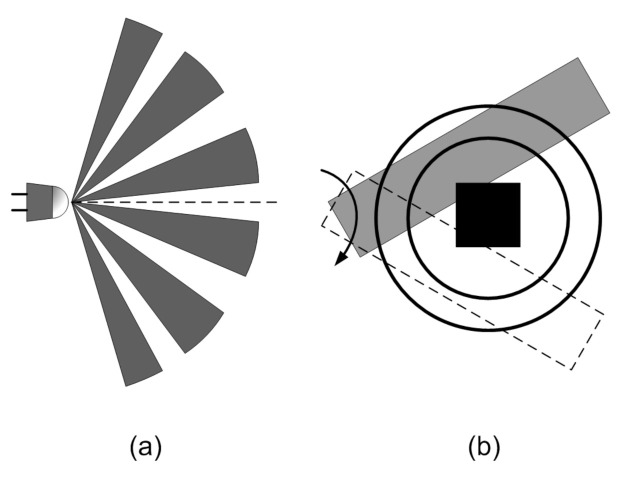
Schematic representation of (**a**) optical and (**b**) mechanical chopping for pyroelectric detectors.

Pyroelectric sensors are sensitive only to the variation of incident IR radiation: they are, therefore, particularly suited as short-range motion detectors, for example for appliances automation (e.g., sanitizing gel dispensers). However, they cannot be employed for realizing contactless human body temperature measurements and, in order to perform presence detection of stationary subjects, some additional expedient, such as optical and mechanical chopping, which are schematically illustrated in Figure 8, must be employed.

Optical chopping [46] employs an array of Fresnel lenses in order to divide the sensor field-of-view into several optically separated cones: in this way, a subject moving from one cone to the other can be detected; otherwise, as a subject moves through the FOV of the sensor, especially if it covers a wide area, only negligible changes in input IR radiation would be sensed. In alternative, a matrix of detectors can be employed, each covering a portion of the overall sensor FOV [47].

Mechanical chopping [48,49,50], instead, employs a shutter to modulate the radiation received by the sensor. The shutter must be moved: a motor, therefore, is needed, thus determining a significant increase to the intrinsic sensor power consumption.

Hence, both optical and mechanical chopping enhance the system complexity, thus increasing its cost and reducing its appeal for implementing applications different from motion sensing.

### 3.3. Thermopiles

Thermopiles are the simplest and most inexpensive thermal detectors. As they self-generate the output voltage signal, in fact, they do not require any biasing: therefore, they are particularly suited for low power applications and allow avoiding self-heating effects. Furthermore, thermopile sensors can be fabricated employing standard CMOS processes, thus enabling low-cost large-volume production. In recent years, hence, much attention has been dedicated to CMOS-compatible thermopile detectors obtained through etching and MEMS post-processing.

For thermopiles, high responsivity and small time constant are desired, in order to obtain a large output signal and a fast response time. Both the responsivity and the time constant depend on the thermocouple leg length: in particular, a large leg length enables high responsivity, while small leg length allows achieving a small thermal resistance and, therefore, a small time constant. Hence, a a compromise must be chosen for the thermopile design, while also taking into account the sensor mechanical stability [51].

An improved mechanical stability and a reduced time constant have been achieved in [51] by replacing a single big thermopile (SBT) structure with a series-connected small thermopile array (STA). The fill factors of SBT and STA are the same, since the small thermopile in STA is scaled down with respect to the SBT. The responsivity and the electrical resistance of SBT and STA are substantially the same; however, the time constant is reduced by 37% for STA with respect to the SBT case.

The responsivity value, however, is not particularly high (∼37 V/W) as the conductor materials employed for the thermocouple elements are polysilicon (poly-Si) and aluminum (Al). Although poly-Si/Al thermocouples have been often employed for their ease of fabrication, they suffer, in fact, low responsivity since aluminum features a low Seebeck coefficient.

**Figure 9:**
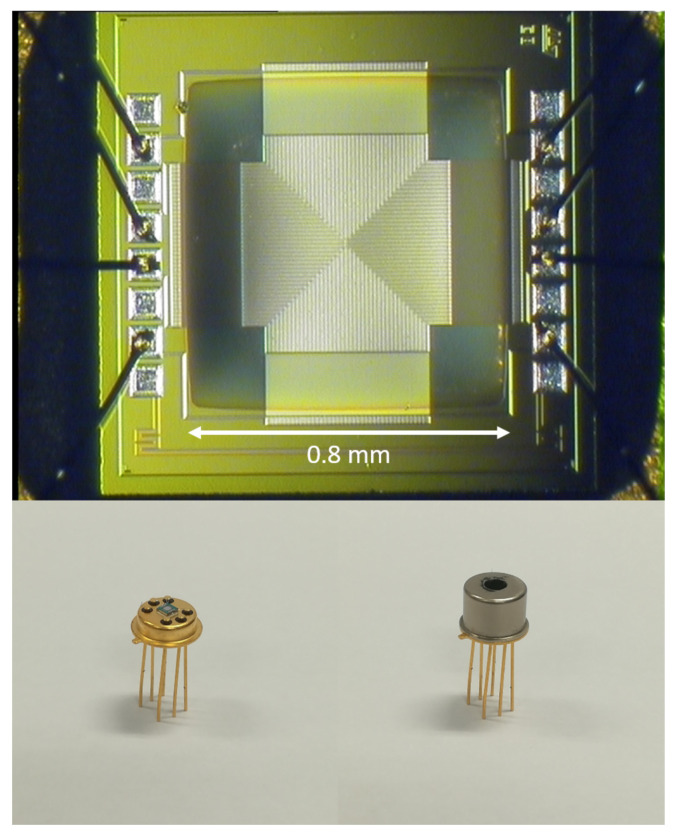
Photograph of the thermopile sensor presented in [15].

In order to achieve higher responsivity values, different materials have been investigated and the focus has been centered, in particular, on polysilicon (poly-Si). The Seebeck coefficients of semiconductors, in fact, are typically much higher than those of metals; furthermore, the Seebeck coefficient of a semiconductor can be adjusted by varying its doping concentration [52,53]. P-poly-Si/N-poly-Si thermopile sensors have been proposed in [15]. The thermopile illustrated in [15], featuring 160 thermocouple elements and a co-planar structure, achieves very high responsivity value in air (180 V/W), while maintaining a limited time constant. This thermopile sensor is illustrated in Figure 9.

In order to further increase the detector responsivity, vacuum packaging may be employed: in [53] a 88.5-V/W responsivity in air is improved to 202.8-V/W under vacuum.

**Figure 10:**
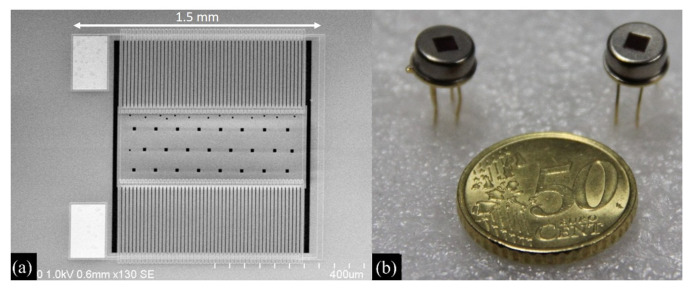
(**a**) Microphotograph of the thermopile sensor and (**b**) photograph of the packaged device presented in [54].

Furthermore, responsivity can be increased by employing double layer structures [53,54]: in this way, the limit imposed by the maximum number of thermocouples that can be fabricated per unit area can be overcome. In a double layer structure the N-type and the P-type thermocouple legs are located in different planes, thus allowing reducing the detector size, while maintaining high performance. The thermopile sensor proposed in [54] is illustrated in Figure 10.

Although polysilicon features a high Seebeck coefficient, it suffers from high thermal conductivity. This drawback, however, is tolerated as polysilicon can be easily manufactured employing CMOS processes, thus determining a significant cost reduction.

Furthermore, reducing the thermal conductance can be achieved, for example, by patterning the thermocouple legs with a phononic crystal structure, as proposed in [55], where 200-nm holes, fully compatible with submicron lithography tools, are etched into the polysilicon.

**Figure 11:**
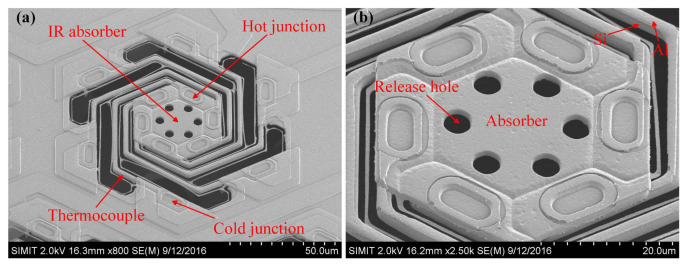
Microphotographs ((**a**) original and (**b**) zoomed version) of the thermopile sensor presented in [56].

In order to achieve higher Seebeck coefficients in semiconductors, their impurity doping concentrations is decreased: this, however, determines an increase of resistivity. Since the thermopile resistance relates to the minimum detectable signal level because of the thermal noise, clearly it is necessary to optimize the impurity concentration in order to obtain a compromise between Seebeck coefficient and resistance for achieving a satisfactory signal-to-noise ratio [56]. As Single-crystalline silicon (SC-Si) features a significantly higher Seebeck coefficient and lower resistivity with respect to polysilicon at the same doping level, SC-Si has been employed in [52,56], together with aluminum, as thermocouple material. The obtained responsivity value is significantly larger than typical poly-Si-based thermopiles, while also featuring a very limited size thanks to its symmetrical helical structure. This thermopile sensor is illustrated in the microphotograph of Figure 11.

Another way to improve the thermopile performance is increasing its absorptivity: this has been achieved by adopting thin dielectric films with good absorption properties or by coating nanomaterials (e.g., porus black materials, metamaterials [57,58]) on the IR absorber surface. The first solution achieves limited absorptivity values (∼0.7 in the 9–14 µm range), while the second suffers from process incompatibility, high cost and high thermal conductivity of metal black materials [59]. A novel strategy to improve the detector absoptivity is illustrated in [59,60]. Pyramidally-textured structures are formed in-situ in the thin dielectric film employed as IR absorber: in this way, the thermopile thermal conductance is reduced and the absorptivity is increased, thus improving the sensor responsivity, while keeping the detector time constant within a few ms. The responsivity values increase from 55.8 to 76.8 V/W and from 91.8 to 147.2 V/W in [59,60], respectively, depending on the different textured/suspended dielectric film ratio.

Another way to enhance the IR absorption efficiency is employing sub-wavelength hole structures in the absorbing area. In [61], a matrix (or square) arrangement (MA) and a staggered (or hexagonal) arrangement (SA) of sub-wavelength rectangular-hole arrays have been employed, giving rise to an IR absorption efficiency 2.6–3.3 times larger with respect to the one of a thermopile with standard absorbing area.

**Figure 12:**
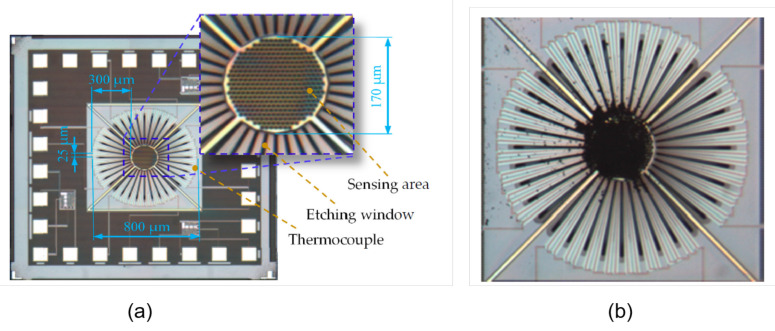
Microphotograph of the thermopile sensor presented in [62] (**a**) before and (**b**) after rGO drop-coating.

Furthermore, the IR absorption efficiency, and therefore the responsivity, could be increased by employing different materials for the absorbing membrane. For example, in [62] reduced graphene oxide (rGO) is deposited by drop-coating on the absorbing area: in this way, the responsivity of the considered thermopile sensor is increased by about 77% with respect to the sensor without the rGO. The considered thermopile sensor is illustrated in Figure 12.

Table 2 reports the main characteristics of different thermopile-based thermal detectors. The considered thermopile sensors have been proposed in the referenced papers for various applications: contactless human body temperature measurements [15,60], motion and presence detection [63], high resolution imaging applications [56], high-precision IR detection applications [54], long-distance measuring thanks an intrinsically reduced FOV angle [62]. Thermopile sensors, therefore, appear particularly well suited for COVID-19 related applications as they are very versatile and can be employed both for contactless fever measurements and for presence and motion detection applications enabling people counting or appliances automation.

### 3.4. TMOS

**Figure 13:**
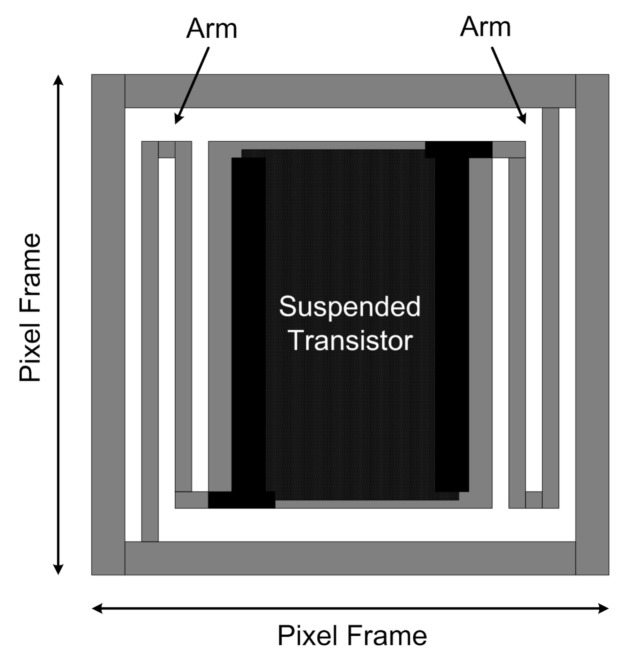
Schematic representation of the TMOS sub-pixel.

The TMOS is based on a CMOS-SOI transistor subjected to MEMS post processing. The TMOS features a mosaic structure as it is composed by several sub-pixels, which are electrically connected, either in parallel or in series or in a combination of the two, while being thermally isolated [25]. The mosaic structure enables enhanced performance and robust wafer level manufacturing. The sub-pixel schematic view is illustrated in Figure 13: the micromachined transistor is embedded on the suspended absorbing membrane, which determines the sensor thermal capacitance, $C_{th}$, while being thermally isolated from the frame by two folding arms, which determine the sensor thermal conductance $G_{th}$.

The TMOS is fabricated by means of built-in masks and dry bulk micromachining: the upper metallization layers typical of standard CMOS processes, made of aluminum or copper, in fact, provide masking, as they are unaffected by the fluorine plasma, which is applied to the dry etching of the silicon and the interlevel dielectrics. The use of built-in masks ensures alignment accuracy and resolution provided by the CMOS processes, while significantly reducing the fabrication cost, thus making the TMOS particularly appealing for consumer applications. In order to improve its efficiency, the TMOS is packaged under vacuum, thanks to wafer level packaging: in this way, air conduction is reduced and the sensor is protected from moisture [24].

**Figure 14:**
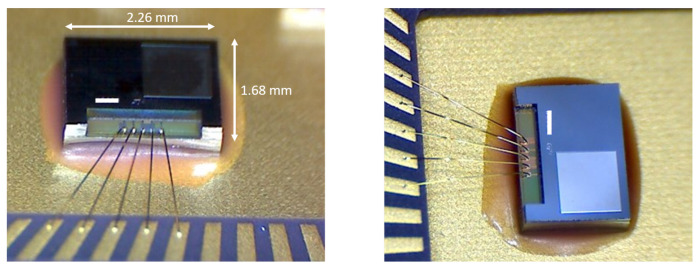
Microphotograph of a packaged TMOS sensor.

Each TMOS package, illustrated in the photographs of Figure 14, contains two TMOS devices, one “active”, exposed to the thermal radiation from the surroundings, and one “blind”, which “sees” only itself as it is shielded from the incoming radiation by an aluminum mirror deposited on the package [24,64]. The two devices are employed in a differential configuration [26,29]: in this way the blind TMOS acts as reference, canceling out the common mode signal and self-heating effects.

Employing the TMOS as a 3-terminal device or in a 2-terminal (diode-like) configuration, TCC and TCV values that well compare with the TCR of bolometers are obtained [64], in particular TCC values above 4%/K are achieved with the TMOS operating in subthreshold region [23]. While the TCR of bolometers suffers from large manufacturing non-uniformity, this is not the case for the TMOS. Furthermore, the TMOS features the advantage of having a very small temperature dependence of the TMOS TCC and TCV for a specific operating point, about 0.02%/K^2^.

Table 3 reports the main TMOS characteristics: being an active device, thanks to its internal gain, very large responsivity and sensitivity values are achieved.

TMOS sensors have already been employed for intruder detection applications [64,65], therefore they can be used for motion and presence detection for appliances automation and occupancy detection. Furthermore, thanks to its high responsivity and sensitivity, the TMOS is very suitable for human body contactless temperature measurements. Moreover, as the latest TMOS version is implemented in 8-inch wafers in 130-nm CMOS-SOI technology, employing wafer level processing in standard CMOS fabs, it can be fabricated at low cost and in large volumes, thus resulting particularly suited for consumer applications.

## 4. Conclusions

All the presented sensor types can be effectively employed as thermal detectors: each type features its own advantages and drawbacks.

Bolometers can achieve very high responsivity, ranging from 1.5 × 10^3^ to 1.5 × 10^5^ V/W, as well as significant TCR values, around 2–4%/K for typical commercial microbolometers and up to 10–29% employing newly investigated materials. Although they clearly feature high performance characteristics, they suffer from the significant disadvantage of requiring medium-high fabrication cost. Nevertheless, in recent years, microbolometers requiring less expensive production steps, at the cost of lower performance, have been investigated. Bolometers can be successfully employed for contactless temperature measurements as well as for motion and presence detection, required for people counting and appliances automation, in consumer electronics; however, their employment is nowadays more spread in military, space and thermography applications, where the cost is secondary with respect to achieving very high performance.

Pyroelectric detectors are quite inexpensive when employed for motion detection and, therefore, particularly suited for appliance automation, such as, for example, non-contact sanitizing gel dispensers. Furthermore, they can easily be used for motion detection applications in indoor environments as they feature a 2–15 m detection range [47]. However, they would require optical or mechanical chopping for temperature measurements and presence and occupancy detection, which would give rise to a significant cost increase.

Thermopile detectors, instead, are very versatile and can be successfully employed both for contactless temperature measurements [15,60] as well as for applications requiring motion or presence sensing, enabling for example occupancy detection or people counting [63,66]. Commercially available thermopiles are already employed in IR thermometers [8,9,10,11] and social distancing applications [67]. Thermopiles feature low responsivity values, ranging from 30 to 1150 V/W: this drawback, however, is compensated by the very low fabrication cost, which makes thermopile detectors the thermal detector of choice for consumer electronics.

In the last ten years, however, the TMOS has emerged, combining low fabrication cost, typical of thermopiles, and high performance, typical of bolometers. The TMOS, in fact, is fully compatible with CMOS processes and features 1.25 × 10^7^ V/W responsivity and 4 %/K TCC. The TMOS, therefore, looks particularly promising for fever detection and social distancing oriented applications.

Thermal detectors, therefore, represent an important resource in the fight against the COVID-19 pandemic as they can be successfully employed in a wide range of applications: from contactless temperature and fever measurements, to motion and presence based detection applications to implement social distancing (e.g., occupancy detection, people counting, non contact appliances operation).

## Figures and Tables

**Table 1:** Comparison between different bolometer detectors.

Parameter	[30]	[32]	Conventional [31]	Meshed Leg [31]	Extended Leg [31]	[33]	[37]	[44]
Material	Vanadium Oxide	Amorphous-Si	Amorphous-Si	Amorphous-Si	Amorphous-Si	Aluminum (CMOS)	ZnO	Cytochrome C
N of thermocouples	80	320	160	96	NA	32	6	184
Responsivity [V/W]	NA	10 × 10^4^	NA	NA	NA	1.751 × 10^3^	NA	1.5 × 10^5^
TCR [%/K]	−2.7	NA	NA	NA	NA	0.385	−10.5	29
Time Constant [ms]	NA	NA	10.68	16.66	4.39	5.27	0.24	NA
$G_{th}$ [W/K]	NA	NA	3.7 × 10^−8^	2.4 × 10^−8^	9.1 × 10^−8^	1.41 × 10^−6^	NA	NA

**Table 2:** Comparison between different thermopile detectors.

Parameter	SBT [51]	STA [51]	[15]	[53]	[54]	[56]	[60]	[62]
Material	n-poly Si/Al	n-poly Si/Al	n-poly Si/ p-poly Si	n-poly Si/ p-poly Si	n-poly Si/ p-poly Si	SC Si/ Al	p-poly Si/ Al	n-poly Si/Al
Responsivity [V/W]	38.66	36.37	180	202.8	1151.14	342	147.2	14.522
Output Resistance [kΩ]	173	176	540	458.5	NA	29	541	11.36
Time Constant [ms]	5.3	3.3	13	NA	14.46	0.56	9.44	3.3
Medium	air	air	air	vacuum	vacuum	air	air	air

**Table 3:** TMOS Characteristics [23,26,64].

Parameter	Value
Current Sensitivity [nA/K]	37
Current Responsivity [A/W]	0.125
Voltage Responsivity [V/W]	1.25 × 10^7^
Time Constant [ms]	80
TCC [%/K]	∼4
